# Comparative Transcriptome and Weighted Gene Co-expression Network Analysis Identify Key Transcription Factors of *Rosa chinensis* ‘Old Blush’ After Exposure to a Gradual Drought Stress Followed by Recovery

**DOI:** 10.3389/fgene.2021.690264

**Published:** 2021-07-15

**Authors:** Xin Jia, Hui Feng, Yanhua Bu, Naizhe Ji, Yingmin Lyu, Shiwei Zhao

**Affiliations:** ^1^Beijing Key Laboratory of Ornamental Germplasm Innovation and Molecular Breeding, China National Engineering Research Center for Floriculture, College of Landscape Architecture, Beijing Forestry University, Beijing, China; ^2^Beijing Key Laboratory of Greening Plant Breeding, Beijing Institute of Landscape Architecture, Beijing, China

**Keywords:** drought stress, comparative transcriptome analysis, weighted gene co-expression network analysis, rose, transcription factors

## Abstract

Rose is one of the most fundamental ornamental crops, but its yield and quality are highly limited by drought. The key transcription factors (TFs) and co-expression networks during rose’s response to drought stress and recovery after drought stress are still limited. In this study, the transcriptomes of leaves of 2-year-old cutting seedlings of *Rosa chinensis* ‘Old Blush’ from three continuous droughted stages (30, 60, 90 days after full watering) and rewatering were analyzed using RNA sequencing. Weighted gene co-expression network analysis (WGCNA) was used to construct a co-expression network, which was associated with the physiological traits of drought response to discovering the hub TFs involved in drought response. More than 45 million high-quality clean reads were generated from the sample and used for comparison with the rose reference genome. A total of 46433 differentially expressed genes (DEGs) were identified. Gene Ontology (GO) term enrichment and Kyoto Encyclopedia of Genes and Genomes (KEGG) analysis indicated that drought stress caused significant changes in signal transduction, plant hormones including ABA, auxin, brassinosteroid (BR), cytokinin, ethylene (ET), jasmonic acid (JA) and salicylic acid (SA), primary and secondary metabolism, and a certain degree of recovery after rewatering. Gene co-expression analysis identified 18 modules, in which four modules showed a high degree of correlation with physiological traits. In addition, 42 TFs including members of *NACs, WRKYs, MYBs, AP2/ERFs, ARFs*, and *bHLHs* with high connectivity in navajowhite1 and blue modules were screened. This study provides the transcriptome sequencing report of *R. chinensis* ‘Old Blush’ during drought stress and rewatering process. The study also identifies the response of candidate TFs to drought stress, providing guidelines for improving the drought tolerance of the rose through molecular breeding in the future.

## Introduction

Rose is one of the most fundamental ornamental crops. With an important economic, cultural, and symbolic value, the rose has a significant market share in the floriculture industry. Cut rose flowers account for 32% of the world’s total cut flower trade. The rose occupies nearly a quarter of the Netherlands floriculture market (Mordor Intelligence, 2020). However, their growth conditions are closely related to changes in the external environment. Most roses suffer from various degrees of drought, which result in a sizable loss of productivity and quality around the world. As a kind of abiotic stress, drought is becoming an even more serious issue due to global warming. Changes in the earth’s climate have an adverse impact on plant growth, crop production, and distribution (Zhu, 2002). It is therefore pressing to identify the response mechanism in rose under the drought stress.

To resistance drought stress, plants have evolved several adaptive mechanisms depending on the activation of the molecular networks involved in stress perception, signal transcription and transduction, expression of specific stress-related genes, and physiological reaction of tolerance (Chaves et al., 2003). Damage to plants under drought stress is manifested in the peroxidation caused by the accumulation of active oxygen in the plant (Niu et al., 2013). The role of various antioxidant protection enzymes in plants under drought stress has been extensively studied. In addition, malondialdehyde (MDA) is an important indicator of cell stability (Fang et al., 2018). Studies have shown that drought stress leads to different trends in plant antioxidant systems (Zandalinas et al., 2017; Niu et al., 2018). The activity of four antioxidant enzymes fluctuate while the MDA content continues to increase in Herbaceous peony with drought stress (Niu et al., 2018). Drought stress accumulates amounts of osmotic adjustment substances in plants such as proline and soluble sugars. In research on the drought stress of rose, it was found that as the drought stress deepened the content of proline and soluble sugar increased (Gadzinowska et al., 2019; Adamipour et al., 2020; Al-Yasi et al., 2020). It is crucial to identify the genes and biological pathways involved in drought stress tolerance and improve the understanding of the molecular mechanisms relating to drought resistance in plants (Valliyodan and Nguyen, 2006). A large number of signaling metabolic pathways and numerous genes have been determined in several model plants exposed to drought environments, such as Arabidopsis (Huang et al., 2018), rice (Liu et al., 2018), and so on. Transcription factors (TFs) control the regulating downstream stress response genes that play essential roles in various abiotic stress response processes (Joshi et al., 2016). During signal transduction, TFs directly regulate the expression of related genes by acting as a molecular switch. These TFs interact specifically with *cis*-elements located in the promoter region of the regulator (Franco-Zorrilla et al., 2014). As the plant drought tolerance network is extremely complex, this study started by exploring the transcriptional regulatory network of TFs for drought resistance. To date, *RcLEA* (Zhang et al., 2014), *RcMYBPA2* (Li et al., 2019c), *RhMYB96* (Jiang et al., 2018), *RcNAC3* (Jiang et al., 2014), and *RcNAC31* (Ding et al., 2019) genes have been reported to improve the abiotic stress resistance of the transgenic plant. However, information on the mechanism of rose response to drought stress is still limited.

As a deep sequencing technology, transcriptome technology has been successfully used to explore the gene network of non-model plant species in response to drought stress. Drought-responsive transcriptome studies have been conducted in ornamental plants such as chrysanthemum (Xu et al., 2013), tree peony (*Paeonia section* Moutan DC.) (Zhao et al., 2019a), and *Dianthus spiculifolius* (Zhou et al., 2017). Previously, only a suppression subtractive hybridization library was constructed for the dehydration stress of cut rose (*Rosa hybrida* cv. Samantha) petals, which contained 3513 unique expression sequence tags, and its expression profile during the dehydration cycle was analyzed (Dai et al., 2012). Although the transcriptome of *R. chinensis* ‘Mutabilis’ in the three stages of drought stress has been reported (Li et al., 2021a) and provided some basis, drought stress generally happens continuously, and it is more essential to discover how the rose recovers after drought stress. In other words, knowledge about the gene co-expression network involved in rose drought and recovery is very limited. WGCNA has become an important tool to identify gene co-expression related to associating with function, which has been widely used in the research of horticultural plants (Lu et al., 2019; Liu et al., 2021).

*Rosa chinensis* ‘Old Blush’ originated from China and has participated in modern rose hybrid breeding. *R. chinensis* ‘Old Blush’ is the diploid species in Rosa, which may represent a convenient genomic model for Rosa research and may be used as a model plant to study rose response to drought stress. Currently, the genome of *R. chinensis* ‘Old Blush’ was published, providing a valuable database for further function and genomic research in rose (Raymond et al., 2018). To obtain unique insights into the molecular mechanisms at the transcriptome level in response to drought and recovery, this study compared transcriptome profiles of *R. chinensis* ‘Old Blush’ at continuous drought stages (30, 60, 90 days after full watering) and rewatering, and constructed co-expression modules using WGCNA. The Gene Ontology (GO) and Kyoto Encyclopedia of Genes and Genomes (KEGG) analysis in differentially expressed genes (DEGs) were performed on the modules with significant physiological data and the key TFs in the module were identified. The study aims to obtain an understanding of the drought responsive regulatory network at the molecular level and identify the key TFs. Furthermore, the results of this study can provide the basis for a better understanding of not only other *R*. *hybrida* cultivars but also other woody plants responding to drought stress, and odder candidate genes for further rose resistance research.

## Materials and Methods

### Plant Materials

Two-year-old healthy cutting seedlings of the *R. chinensis* ‘Old Blush’ used in the experiments were collected from the Beijing Institute of Landscape Architecture (Beijing, China). The plant material was planted in the pot with an inner size of 26 cm in diameter, 22 cm in height, and 18 cm in diameter at the bottom. It contained sterilized soil with a mixture of nutritive soil, garden soil, perlite, and vermiculite with the ratio of 5:3:1:1 (v/v). The size and height of plant materials used in all experiments were almost the same. Before drought stress, samples were cultured under the following growth conditions: 50% relative humidity, 25°C/18°C, day/night temperatures in the same artificial climate chamber in Beijing Forestry University (BJFU) (116.3°E, 40.0°N). Then, the samples were subjected to a continuously dry environment (no-watering) for 15, 30, 45, 60, 75, and 90 days. Then, after 7 days with rewatering treatment, the leaves were totally recovered. Fully watered plants were used as a control. Refer to experimental methods of drought treatment from David Gamboa-Tuz et al. (2018). At each time point, the fourth or fifth fully expanded leaves from the plants were collected, then immediately frozen in liquid nitrogen and stored at −80°C until the physiological measurement or RNA extraction. Each treatment group contained three replicates, and each sample from the three replicates was used to obtain biological replicates.

### Measurement of Physiological Parameters

Before transcriptome sequencing using leaf tissue, which was collected and stored at −80°C from plants subjected to 0 to 90 days of drought stress, the relative water content of leaves, superoxide dismutase (SOD) activity, MDA, and relative soil water content (RSWC) was evaluated. RSWC was analyzed using direct drying method. The relative water content of leaves was determined by the initial fresh weight of the leaf samples, referencing from Zhou et al. (2019) with slight modifications, the samples were soaked in distilled water placed on the lab bench with dark cloth for 24 h and finally weighted, The thiobarbituric acid (TBA) reaction was used for evaluation of the MDA content as described by Heath and Packer (1968) with minor adjustments. The SOD activity was measured after the spectrophotometer by determining its ability to inhibit the photochemical reduction of nitro blue tetrazolium (NBT). Each experiment included three biological replicates. The correlation analysis for various physiological parameters used the Pearson correlation coefficient in SPSS software.

### RNA Isolation, cDNA Library Preparation, and Transcriptome Sequencing

According to the manufacturer’s instructions, total RNA was isolated with an Easy Spin Plus RNA Extraction Kit (RN53, Aidlab China). RNA integrity was verified using electrophoresis on 1% agarose gel. The 2100 Bioanalyzer (Agilent Technologies, United States) and ND-2000 (NanoDrop Technologies) were employed to test the quality and purity of RNA.

The RNA-Seq library was generated using the NEB#7530 RNA Library Prep Kit (#E7530, New England Biolabs). The total mixed RNA from *R. chinensis* leaves in all treatments was used for cDNA library construction. The library quality was subsequently detected through the High Sensitivity DNA assay Kit (Agilent Technologies). The three biological replicates from each experiment were sequenced using an Illumina HiSeq^TM^ 4000 platform by Genedenovo Biotechnology, Co., Ltd. (Guangzhou, China).

To investigate genes involved in the transcriptome, a cDNA sample was prepared from an equal mixture of t all RNA. The total RNA was isolated from the leaves of 2-year-old cutting seedlings *R. chinensis* from the control, drought-treated (30, 60, 90 days) and rehydration groups, designated as CK1, CK2, CK3, DT1-1, DT1-2, DT1-3, DT2-1, DT2-2, DT2-3, DT3-1, DT3-2, DT3-3, RW1, RW2, RW3. Three biological replicates from each group were analyzed on the Illumina HiSeq 4000 platform.

To get high quality clean reads, we were further filtered by fastp (version 0.18.0). The parameters were as follows: first removing reads containing adapters, and then removing reads containing more than 10% of unknown nucleotides (N), finally, removing low quality reads containing more than 50% low quality (*Q*-value ≤ 20) bases.

### Mapping to the Rose Genome and Gene Expression Quantification

HISAT2.2.4 software (Kim et al., 2015) was used to carry out comparative analysis based on the rose genome (Raymond et al., 2018). The comparison results of HISAT2 comparison StringTie (Pertea et al., 2015, 2016) were used to reconstruct the transcript, and the expression of all genes in each sample was calculated.

### Identification of DEGs and Genes Co-expression Network Analysis

The Fragments Per Kilobase of Exon Per Million Mapped Fragments (FPKM) method was used to analyze the expression level of each transcript. The reference-based approach was used to map the reads of each sample assembled by StringTie v1.3.1. For each transcription region, an FPKM value was calculated by StringTie software to quantify its expression abundance and variations. Differential expression analysis was performed by DESeq2 software (Love et al., 2014) between two different groups [also by edgeR (Robinson et al., 2010) between two samples]. The genes with the parameter of false discovery rate (FDR) below 0.05 and absolute fold change ≥ 2 were considered DEGs. GO enrichment analyses of DEGs (Ashburner et al., 2000) were able to recognize the main biological functions. Then, KEGG (Ogata et al., 1999) pathway enrichment analysis identified significantly enriched metabolic pathways or signal transduction pathways in DEGs.

Co-expression networks were established using WGCNA (v1.47) package in R (Langfelder and Horvath, 2008). Gene expression values were imported into WGCNA to construct co-expression modules using automatic network construction function blockwise Modules with default settings, except that the power was 10, TOMType unsigned, and minModuleSize was 50. The correlation coefficient between module eigengenes and physiological data was calculated to find out significant modules, and the hub TFs were selected from those modules (Huang et al., 2020). The networks were visualized using Cytoscape_3.3.0 (Shannon et al., 2003), GO and KEGG enrichment analyses were conducted for genes in each module. The calculated p-value was subjected to FDR and correction, taking FDR ≤ 0.05 as a threshold.

### Real-Time Quantitative PCR Verification

Total RNAs were isolated from the leaves of the treatment and controlled specimens as described above. According to the manufacturer’s instructions, first-strand cDNA synthesis was performed using Prime Script II 1st strand cDNA Synthesis Kit (Takara, Shiga, Japan). Based on the manufacturer’s protocol, the qRT-PCR was performed using a Bio-Rad/CFX Connect^TM^ Real-Time PCR Detection System (Bio-Rad, CA, United States) with SYBR^®^ qPCR mix (Takara, Shiga, Japan). Relative mRNA content was calculated using the 2^–△△Ct^ method against the internal reference gene *RcPP2A* (Klie and Debener, 2011). The primers used in this study were designed with Primer Premier 5 and are listed in Supplementary Table 12. Three biological replicates were performed for all reactions.

## Results

### Changes in Phenotype and Physiology at Drought Stress in *R. chinensis*

**FIGURE 1:**
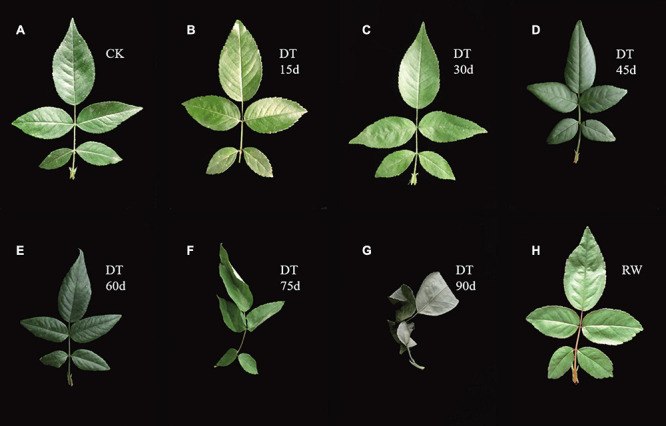
Leaf phenotypes of *Rosa chinensis* ‘Old Blush’ leaves during drought and rewatering stages. **(A)** The control plants. **(B)** Drought treatment for 15 days. **(C)** Drought treatment for 30 days. **(D)** Drought treatment for 45 days. **(E)** Drought treatment for 60 days. **(F)** Drought treatment for 75 days. **(G)** Drought treatment for 90 days. **(H)** Rewatering treatment.

**FIGURE 2:**
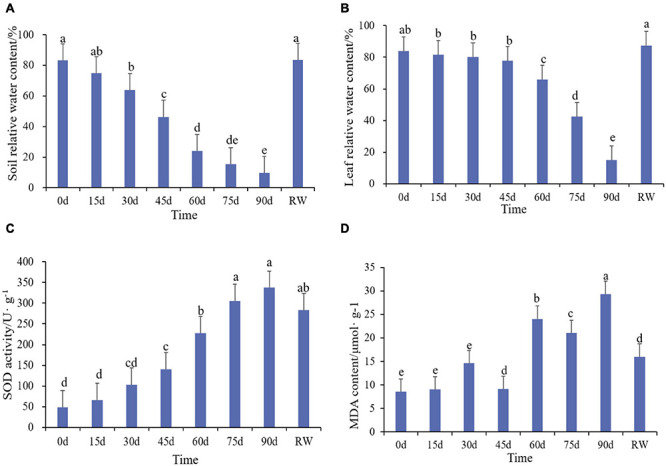
Physiological and biochemical changes of *Rosa chinensis* ‘Old Blush’ under drought and rewatering treatment. **(A)** Soil relative water content. **(B)** Leaf relative water content. **(C)** MDA content. **(D)** SOD activity.

The experiment applied drought stress by stop watering. Plant leaf phenotypes were recorded and physiological indexes were measured under control condition (CK) at 15, 30, 45, 60, 75, 90 days after stress and rewatering. Leaves were dark green and shiny during normal conditions (Figure 1A). After 15 days of drought treatment, the leaves has no obvious morphological changes (Figure 1B). Similarly, the RSWC and leaf relative water content were not significantly different from the control (Figures 2A,B). At 30 days the leaves were slightly wrinkled (Figure 1C). When drought stress lasted for 45 days and 60 days, the leaves were all curled (Figures 1D,E). At this time the relative water content of the soil considerably decreased, reaching a moderate degree of stress (Figure 2A). At 60 days, the relative water content of the leaves decreased significantly compared with the control, which was consistent with the phenotype (Figure 2B). The curl degree of the leaf deepened and the leaf became thinner, and the relative water content of the leaf has a material decrease during 75 days of drought (Figures 1F, 2B). Both leaf relative water content and soil relative water content reached the lowest value, and the leaves were severely curled and withered after 90 days of drought (Figures 1G, 2A,B). After rewatering, the leaves were green and thin (Figure 1H). In order to determine the exact point for transcriptome sequencing, physiological indicators were also measured. The MDA content of leaves treated with drought and rewatering was determined. With the deepening degree of drought stress, the content of MDA rises in fluctuation and reached the highest point after 90 days of the drought treatment (Figure 2D). In addition, SOD increased with the increasing of drought treatment time and achieved the maximum value after 90 days (Figure 2C). Both MDA and SOD content increased significantly during drought treatment for 30 days, 60 days, and 90 days. We conducted correlation analysis on physiological data. MDA content and SOD activity were positively correlated with different treatments and were significantly correlated at the 0.01 level (0.885, 0.979), while the relative leaf water content and soil relative water content were negatively correlated, at 0.01 significantly correlated horizontally (−0.891, −0.987). After rewatering, the MDA and SOD contents were all reduced. Based on these results, leaves were selected under normal conditions, drought treatment for 30 days, 60 days, 90 days, and rewatering treatment for transcriptome sequencing.

### Transcriptome Analysis of *R. chinensis*

The clean reads were obtained by removing adaptor sequences, ambiguous nucleotides, and low-quality reads (*Q*-value < 20), which ranged from 41397356 to 54756474. The Q20 and Q30 values of all 15 libraries were more than 97% and 92%. The GC content was approximately ranged from 48% to 49%. Between 92.33% and 93.89% of the sequenced reads could be aligned to the rose reference genome (Table 1). In this study, the proportion of exon sequences ranged from 95.30% to 96.38%, and the proportion of intron sequences ranged from 1.45% to 2.66% (Supplementary Figure 1).

**TABLE 1:** RNA sequencing data and corresponding quality control.

**Sample name**	**Clean reads**	**Raw reads**	**Mapped reads**	**GC Content (%)**	**Q20 (%)**	**Q30 (%)**
CK1	48126136	48181768	44510062 (93.27%)	48.28	97.51	92.92
CK2	49106032	49156710	45429755 (93.89%)	48.66	98.03	94.17
CK3	43793126	43853234	40291282 (93.01%)	48.29	97.48	92.88
DT1-1	45651270	45706946	42334367 (93.31%)	49.52	97.67	93.28
DT1-2	48888834	48972378	44986749 (92.70%)	48.11	97.38	92.70
DT1-3	53820314	53902378	49546849 (92.94%)	48.30	97.46	92.85
DT2-1	47463436	47521292	43862640 (93.07%)	48.19	98.12	94.31
DT2-2	54353944	54420772	49969726 (93.02%)	48.23	98.10	94.27
DT2-3	51937470	52002482	47795161 (92.33%)	48.09	98.13	94.32
DT3-1	50304612	50359618	40931525 (93.07%)	49.61	98.27	94.71
DT3-2	44365148	44421624	38029228 (93.35%)	49.58	98.10	94.28
DT3-3	49072308	49130228	41525793 (93.40%)	49.60	98.18	94.50
RW1	54756474	54838556	50746877 (93.11%)	48.65	97.99	94.00
RW2	41397356	41446080	38442300 (93.52%)	48.94	97.91	93.88
RW3	48571772	48639152	45164692 (93.24%)	48.56	98.16	94.46

### Comparisons of DEGs Under the Different Drought Stress Stages

**FIGURE 3:**
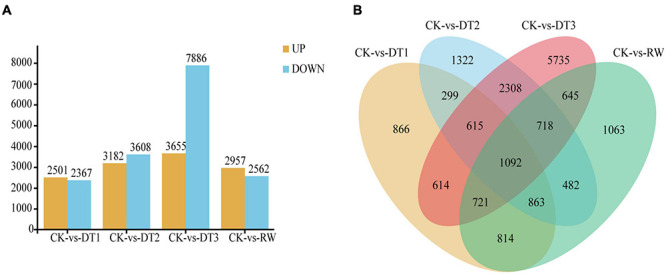
RNA-seq data expression profiles during drought and rewatering treatment in *Rosa chinensis* ‘Old Blush.’ **(A)** The number of genes up-regulated and down-regulated in four drought and rewatering treatment stages compared with the control. Genes up-and down-regulated are shown as yellow and blue bars. **(B)** Venn diagram analysis of the DEGs from four drought and rewatering treatment stages compared with control plants.

To analyze the DEGs between leaves that suffered from drought stress at four different time duration (30, 60, 90 days and rewatering) and the control, comparisons were conducted between five groups. The DEGs were filtrated according to an expression level | log_2_(FC)| > 1 and FDR < 0.05 in each pairwise comparison. 6380 genes were identified exclusively at DT3, while 866 genes were specific in DT1. Especially 6380 (35.14%) genes were identified in DT3, but only 1680 (9.25%) and 1804 (9.94%) genes were identified in DT1 and DT2 (Figure 3B).

The number of DEGs is an increase from DT1 to DT3 and reached a peak at the CK vs. DT3 comparison set, indicating most of the drought regulated genes had a late response. The results showed DEG enrichment in CK vs. DT1, CK vs. DT2, and CK vs. DT3, illustrating the number of down-regulated DEGs was significantly higher than the number of upregulated DGEs. Meanwhile, a total of 11545 DEGs including 7886 downregulated and 3659 upregulated between CK and DT3 which contains the largest number of observations among all drought stress groups. Only 2503 upregulated and 2367 downregulated were observed between CK and DT1, suggesting that the differentiation of expressed genes between CK and DT3 is larger than that of CK and DT1. The result indicates that transcript abundance changed dramatically at key switched among the drought stages, which drought response genes could be induced and expressed largely. Compared with CK, there are slightly more genes upregulated than genes downregulated in RW (Figure 3A).

### GO Functional Enrichment and KEGG Pathway Enrichment Analysis of DEGs

The drought resistance process in *R. chinensis* is complex According to GO functional enrichment analysis, as the drought stress deepened, the most enriched GO category among these DEGs was ‘metabolic process,’ followed by ‘cellular process,’ ‘biological regulation,’ ‘regulation of biological process,’ ‘response to stimulus,’ ‘catalytic activity,’ ‘binding,’ ‘transport activity,’ ‘cell part,’ ‘cell,’ ‘organelle,’ ‘membrane,’ and ‘membrane part.’ Meanwhile, based on the GO analysis of up-regulated DEGs, the GO terms of up-regulated DEGs in CK vs. DT1, CK vs. DT2, and CK vs. RW were mostly concentrated on membranes, including the cellular components category, such as ‘membrane’ (GO:0016020), ‘membrane part’ (GO:0044425), and ‘intrinsic component of membrane’ (GO:0031224) (Supplementary Table 1). CK vs. DT3 also involved more cells and metabolic processes including the biological process category (metabolic process, GO:0008152, cellular, GO:0009987, single-organism process, GO:0044699, cellular metabolic process, GO:0044237) (Supplementary Table 1). Moreover, the GO analysis of the down-regulated DEGs found that among the top 20 enriched, 13 of them belong to the category of biological processes, mainly focusing on the response to stimuli in CK vs. DT1 (Supplementary Table 2). It is worth noting that the GO analysis of DT3 vs. RW mainly focused on cells and cell membranes, indicating that the rose has been restored to a certain extent at the cell level (Supplementary Tables 1, 2).

The KEGG pathway analysis revealed that it is mainly enriched in the metabolism under the different drought degrees and rewatering treatment. Among the top 10 enriched pathways, ‘plant–pathogen interaction’ (ko04626), ‘biosynthesis of secondary metabolites’ (ko01110), ‘plant hormone signal transduction’ (ko04075), ‘MAPK signaling pathway-plant’ (ko04016), were regulated in response to DT1, indicating that drought stress was sensed and signaling transduction pathways were activated. However, more pathways related to metabolism were enriched at DT2 and DT3. The ‘photosynthesis’ (ko00195) and ‘photosynthesis-antenna proteins’ (ko00196) associated with photosynthesis were all enriched in CK vs. DT2 and CK vs. DT3 (Supplementary Table 3). Metabolite-related pathways such as ‘biosynthesis of secondary metabolites’ (ko01110), ‘alpha-Linolenic acid metabolism’ (ko00592), ‘tryptophan metabolism’ (ko00380), ‘glycosphingolipid biosynthesis-lacto and neolacto series’ (ko00601), ‘alanine, aspartate and glutamate metabolism’ (ko00250), ‘galactose metabolism’ (ko00052), and ‘glyoxylate and dicarboxylate metabolism’ (ko00630) were enriched in CK vs. DT2 and CK vs. DT3. These results indicated that the metabolism processes of *R. chinensis* may act as the predominant process in the middle and later stages of drought stress. This result was unsurprising, as drought causes changes in the metabolism of plants to improve their drought tolerance. Meanwhile, secondary metabolism, primary metabolism, and photosynthesis were focused in DT3 vs. RW, indicating that the metabolism and photosynthesis of the rose after rewatering recovered, and that drought stress did not cause serious damage to the rose (Supplementary Table 3). Apart from metabolism enrichment, ‘plant–pathogen interaction’ (ko04626) was also enriched in CK vs. RW, indicating that plants are vulnerable to pathogenic bacteria after recovering from drought stress (Supplementary Table 3).

### Identification of Plant Hormone and Signal Transduction-Related DEGs

In this study, signal transduction in plants was influenced by drought stress which was verified by both the GO and KEGG enrichment analysis of the DEGs. More than 240 genes were predicted to encode protein kinases with varying expression levels in *R. chinensis*. Genes encoding receptor-like protein kinases (*RLKs*), in which LRR receptor kinases were the majority accounted for the largest proportion of these genes. Twenty-nine of them were up-regulated in DT1 and DT2. Among them, *MIK1* (RchiOBHm_Chr6g0286591) was up-regulated 8.02-fold, respectively. More than 66 *RLK* genes were included in the group of serine/threonine-protein kinases during the drought stress. Furthermore, one gene related to G-type lectin S-receptor-like serine/threonine-protein kinase showed the highest degree of upregulation in DT3. In the KEGG pathway analysis of DEGs, the ‘MAPK signaling pathway’ (ko04016) was induced during drought treatment. For DEGs detected in DT1 and DT2, two *MAPKKKs* including RchiOBHm_Chr2g0158821 and RchiOBHm_Chr5g0002581 and four *MAPKKs* including, *MPK9* (RchiOBHm_Chr7g0178621), *MKK10* (RchiOBHm_Chr7g0211301), *MEKK1* (RchiOBHm_Chr7 g0240941), *MPK4* (RchiOBHm_Chr7g0196781) were up-regulated. *MKK10* (RchiOBHm_Chr7g0211301) was particularly up-regulated 9.18-fold. However, three *MAPKKs* consist of *MPK3* (RchiOBHm_Chr5g0061451), *MKK4* (RchiOBHm_Chr2g0150341), and *MEKK1* (RchiOBHm_Chr1g0352161) were down-regulated. Also, *MPK4* (RchiOBHm_Chr7g0196781) and *MPK19* (RchiOBHm_Chr5g0028971) were substantially increased in DT3 (Supplementary Table 4).

Ca^2+^ signal transmission is achieved through the generation, decryption, and transmission of specific calcium signals and the corresponding physiological and biochemical reactions downstream. The study identified DEGs related to calcium ion, encoding calcium-binding proteins, Ca^2+^-binding protein EF hand, calmodulin-like, calcium-dependent protein kinases, calcium-transporting ATPase, and calcium channel protein. One gene (RchiOBHm_Chr2g0158571) encoding the calcium channel protein sustained high expression under drought stress and was up-regulated 6.44-fold in DT1 vs. CK (Supplementary Table 5).

According to the GO and KEGG analyses of the DEGs, the GO term ‘hydrogen peroxide metabolic’ (GO:0042743) and ‘response to reactive oxygen species’ (GO:0000302) were enriched at DT1 and DT2, while peroxisome (ko04146) was induced at DT3. 69 DEGs encoded ROS production and scavenging. A total of 55 DEGs encoding enzymes related to ROS scavenging, including peroxidase (POD), ascorbate peroxidase (APX), glutathione *S*-transferase (GST), glutathione peroxidase (GPX), polyphenol oxidase (PPO), ferritin, glutaredoxin, thioredoxin, and peroxiredoxin. Among these antioxidant enzymes, *POD45* (RchiOBHm_Chr4g0400821) encoding POD was up-regulated by 4.79-fold at DT1, another gene encoding POD *PNC1* (RchiOBHm_Chr5g0072281) has no significant change from DT1 to the control, but 9.29-fold has a significant at DT2, reaching the peak of expression level at DT3 (Supplementary Table 6).

The expression of genes involved in hormone biosynthesis or signaling was substantially changed during the drought stress and rewatering stage. The changed genes included ABA, auxin, BR, cytokinin, ET, JA, and SA. Among these DEGs, most genes were involved in ABA response. Nine-*cis*-epoxycarotenoid dioxygenase (NCED) is a critical enzyme in ABA biosynthesis. For instance, one gene encoding *NCED3* (RchiOBHm_Chr5g0014331) was up-regulated by 2.70-fold at DT1, while another gene encoding NCED6 (RchiOBHm_Chr4g0397001) was significantly down-regulated 9.40-fold at DT3. The expression of a gene (RchiOBHm_Chr5g0049951) encoding *AAO* is also a vital enzyme in ABA biosynthesis, which was up-regulated by 2.78-fold at DT1 and down-regulated by 7.28-fold at DT3. Moreover, some genes involved in the ABA mediated signaling pathway were also induced by drought stress. Abscisic acid receptor *PYL4* (RchiOBHm_Chr1g0371101) was down-regulated. In contrast, one gene encoding *PP2C* (RchiOBHm_Chr5g0066401), a negative regulator of ABA, has an up-regulation (Supplementary Table 7).

A total of 24 genes involved in the auxin signaling pathway changed. Three genes encoding auxin response factors (*ARF3*, *ARF5*, *ARF9*) were drought induced. Particularly, *ARF3* (RchiOBHm_Chr5g0009381) expression continued to decline under drought stress, which was induced 4.41-fold at DT3. At the same time, the expression level of *ARF5* (RchiOBHm_Chr6g0302551) continued to increase under drought stress. Two genes encoding *AUX/IAA* proteins were up-regulated at DT1 and down-regulated at DT3. *AUX22D* (RchiOBHm_Chr4g0389611) was lowered nearly 10-fold at DT3. Furthermore, some genes participated in auxin response and signal, for example of auxin-binding protein ABP19a-like (*ABP19A*), auxin transporter-like protein 2 (*LAX2*), and auxin efflux carrier (*PIN1C*). The gene encoding *ABP19A* (RchiOBHm_Chr2g0094781) was up-regulated 7.41-fold at DT1. Five genes encoding *SAUR* were identified, among these, *SAUR77* (RchiOBHm_Chr5g0026611) was up-regulated 9.78-fold at DT1 (Supplementary Table 7).

In this study, a large number of ethylene-responsive transcription factor (*ERF*) genes displayed a down-regulation trend under early drought stress. However, one gene encoding *ERF023* (RchiOBHm_Chr1g0360021) had an up-regulation 3.70-fold at DT1 and was then down-regulated 5.02-fold at DT3. Another gene encoding example, *ERF110* (RchiOBHm_Chr6g0274591) was up-regulated 7.34-fold at DT3. Meanwhile, ethylene receptor (*ERS1*), ethylene-overproduction protein 1 (*ETO1*), and ethylene insensitive3-like protein (*EIL3*) were identified (Supplementary Table 7).

Most of the genes associated with the biosynthesis or signaling of jasmonic acid (JA) were down-regulated. The gene encoding malate dehydrogenase (*MDHG*) was down-regulated 4.06-fold at DT3, while 12-oxophytodienoate reductase 3-like (*OPR3*) was up-regulated 4.13-fold at DT3. Additionally, the majority of genes were related to cytokinin, salicylic acid, and brassinosteroid pathways. One gene encoding cytokinin dehydrogenase 3 (RchiOBHm_Chr2g0093491) was down-regulated 7.40-fold at DT3 (Supplementary Table 7).

### Expression of Genes Involved in Metabolism and Biosynthesis

The output of GO and KEGG enrichment analysis indicates that many DEGs were related to metabolism and biosynthesis. The ‘polysaccharide catabolic process’ (GO:0000272) and the ‘Starch and sucrose metabolism’ (ko00500) pathway were significantly induced by drought stress. Related to carbohydrate synthase and starch synthase were induced. The expression level of sucrose synthase increased, and one of the genes encoding *SUS7* (RchiOBHm_Chr3g0488651) up-regulated 4.03-fold at DT3. Only a small portion of starch synthase genes were down-regulated, others appeared to be up-regulated. Additionally, drought treatment significantly induced the expression of enzyme genes involved in starch and sugar metabolism, fructose, and mannose metabolism. Among these, genes encoding fructose-1,6-bisphosphatase, fructose-bisphosphate aldolase, mannose-1-phosphate guanyltransferase alpha, pyrophosphate–fructose 6-phosphate 1-phosphotransferase subunit beta-like and 6-phosphofructo-2-kinase/fructose-2,6-bisphosphatase were down-regulated at DT3 (Supplementary Table 8).

**FIGURE 4:**
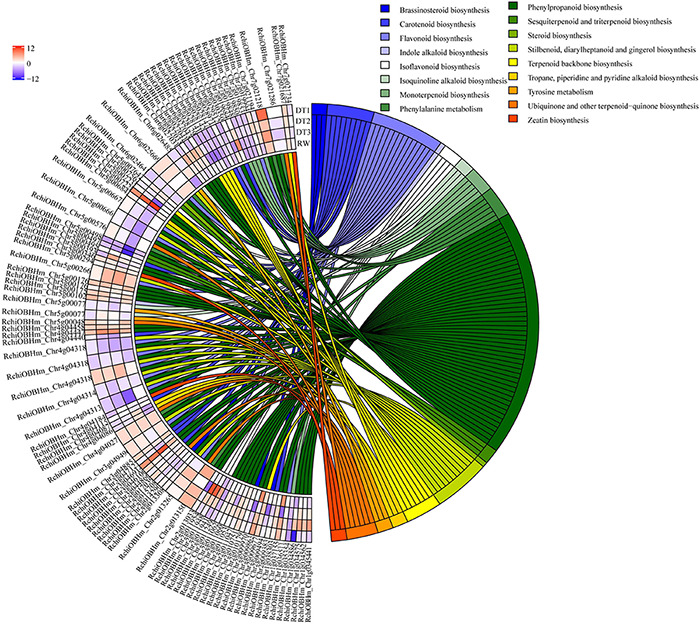
KEGG enrichment of differentially expressed genes involved in secondary metabolism.

In total, 89 DEGs associated with lipid metabolism were enriched in the ‘lipid metabolic process’ (GO:0006629). Most of these were related to the biosynthesis and metabolic processes of lipoprotein, wax, keratin, lipid, and fatty acid. Moreover, most genes involved in wax biosynthesis were significantly up-regulated. For example, one gene encoding *CYP86B1* (RchiOBHm_Chr4g0432631) up-regulated 9.90-fold at DT3 (Supplementary Table 9). The KEGG functional annotation of these differentially expressed genes was related to metabolism and differentially expressed genes were also related to secondary metabolisms. A total of 95 DEGs involved in secondary metabolism were enriched by KEGG function, and the results are shown in Figure 4. Drought stress affected the transcription level of genes that related to phenylpropanoid biosynthesis pathways.

### Photosynthesis-Related Genes Under Drought Stress

Many DEGs enriched in the category of ‘photosynthesis’ (ko00915) were overexpressed in DEGs down-regulated by DT2 and DT3. In the process of DT2 and DT3, many genes involved in photosynthesis were significantly down-regulated. A total of 70 DEGs involved in photosynthesis were identified. In the seam, three (*CAB40*, *LHCB5*, *psaA*) of 16 DEGs encoding the components of photosystem I (PSI) were slightly up-regulated in DT1, and other genes were significantly down-regulated during the drought treatment period. A total of 33 genes encoding PS II were identified, including 6 up-regulated genes (*psbE*, three genes encoding *psbC*, *psbD*, *PSB27-1*), and 27 down-regulated genes. Most of these genes decreased significantly in the middle and later periods of drought, indicating that PS I and PS II were suppressed under drought stress. Meanwhile, there were 15 genes encoding redox chains, all of which were significantly down-regulated in the middle and late periods of drought stress. Additionally, the expression of six gene encoding components of chloroplasts significantly decreased (Supplementary Table 10).

### Transcription Factors Responding to Drought Stress

Transcription factors specifically binding to *cis*-acting regulatory elements in the promoter of target genes are crucial regulatory proteins, which can modulate numbers of genes up or down-regulation (Dhriti and Ashverya, 2015). In this study, *WRKY* (58), *NAC* (43), *MYB* (52), *bZIP* (19), *bHLH* (55) families contained large numbers of transcription factors. The expression level of the *WRKYs* family exhibited large changes under different treatments. The expression of some genes reached a maximum in the middle and later periods of drought. Genes encoding *WRKY75* and *WRKY71* all reached the highest expression level at DT3 with an up-regulation of more than 5-fold. *WRKY57* (RchiOBHm_Chr5g0083891) up-regulated regarding expression responses at different drought stress, especially increased 7.93-fold at DT3. However, more than half of the *WRKY* families have decreased expression levels at different drought levels. Twenty-four genes encoding *WRKYs* were down-regulated at DT1 by 1.02- to 3.71-fold (Supplementary Table 11).

More than half of the genes encoding *NACs* were up-regulated to varying degrees under drought stress. One gene encoding *NAC100* (RchiOBHm_Chr2g0167461) continued to increase in the middle and later periods of drought stress by 7.20-fold. A total of 11 genes upregulated during DT1. Some genes increased and then decreased under drought stress, but finally increased after rewatering. For example, *NAC073* (RchiOBHm_Chr0c19g0500091) was upregulated 5.78-fold at DT1, decreasing slightly at DT2, then down-regulated 2.64-fold at DT3, but had an increase of 6.96-fold after rewatering. *NAC037* (RchiOBHm_Chr2g0099671) had a similar expression trend, but the up-regulation decreased a small amount. On the contrary, one gene encoding *NAC072* (RchiOBHm_Chr5g0034761) was down-regulated at DT1 and continued to increase afterward (Supplementary Table 11).

Under different drought treatments, more than three-quarters of the *MYB* genes’ expression levels showed different degrees of up-regulation. Among them, a relatively large number of up-regulated genes appeared during the DT1 period. For instance, *MYB46* (RchiOBHm_Chr1g0315931) and *MYB61* (RchiOBHm_Chr1g0315931) showed the highest up-regulation of expression with 7.09 and 7.15-fold. Moreover, some genes up-regulated significantly at DT3. *MYB75* (RchiOBHm_Chr2g0116041) displayed the most amount of up-regulation among all up-regulated genes at DT3, with an increase of 7.65-fold. Some genes showed a tendency to increase and then decrease during drought treatment, for example, gene encoding *MYB61* (RchiOBHm_Chr3g0458721) slightly increased at DT1 and then decreased by 6.10-fold. After rewatering, most gene expression did not change significantly compared with CK, only a small amount of gene expression up-regulated (Supplementary Table 11).

Among the three drought treatments of the *bHLH* families, the numbers of genes up-regulated in one period were more than the numbers of genes down-regulated. The number of down-regulated genes in two periods was more than that. Most genes in the *bHLH* family up-regulated during early drought treatment of DT1. While a large number of genes significantly down-regulated during the middle and later drought periods (DT2 and DT3). Among these, there was a slight upward adjustment, up-regulated by 1.02- to 4.66-fold at DT1. Nevertheless, there were 37 genes down-regulated at DT2 or DT3. One gene encoding *bHLH72* (RchiOBHm_Chr7g0182341) down-regulated 6.94-fold. A gene *bHLH121* (RchiOBHm_Chr6g0245181) that belongs to the *bHLH* family has no significant change in gene expression during drought treatment but appeared to be up-regulated after rewatering (Supplementary Table 11).

Among *AP2/DREB* and *bZIP* transcription factors, more genes exhibited down-regulation under drought treatment. Nineteen members of *bZIPs* were found to be responsive to drought stress, while 12 down-regulated from 1.24- to 4.80-fold (Supplementary Table 11). Five genes encoding *DREBs* showed down-regulation that exceeded 6.60-fold at DT1. The most significant down-regulation occurred by *DREB1D* (RchiOBHm_Chr7g0199351), which down-regulated 9.64-fold (Supplementary Table 11).

### Co-expression Network Construction and Identification of WGCNA Modules

**FIGURE 5:**
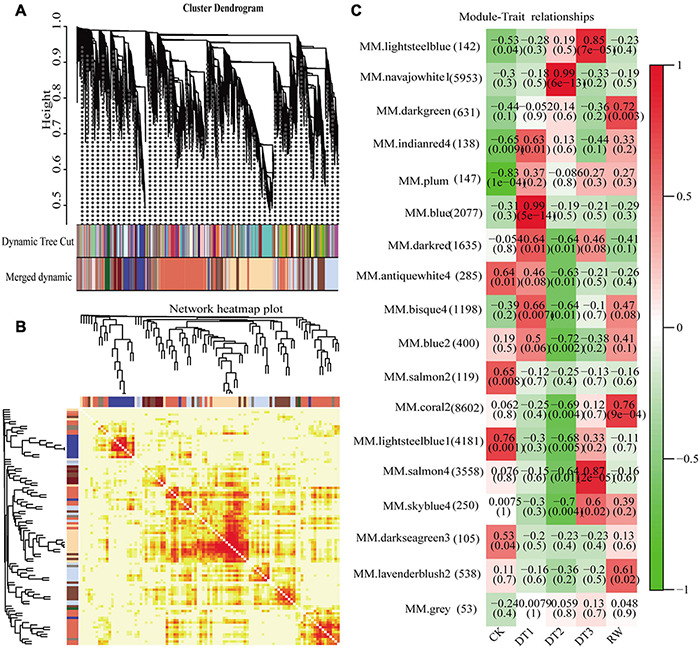
Identification of DEGs by WGCNA. **(A)** Module level clustering diagram. **(B)** Network heatmap plot. **(C)** Module-trait associations. (Each column corresponds to different processing conditions, and each row corresponds to the characteristic gene of the module. The correlation between two is indicated in cell by Pearson correlation coefficient and p value in parentheses. Cell color ranges from red (high positive correlation) to green (high negative correlation), and the number of genes contained in each module is in left parentheses).

**FIGURE 6:**
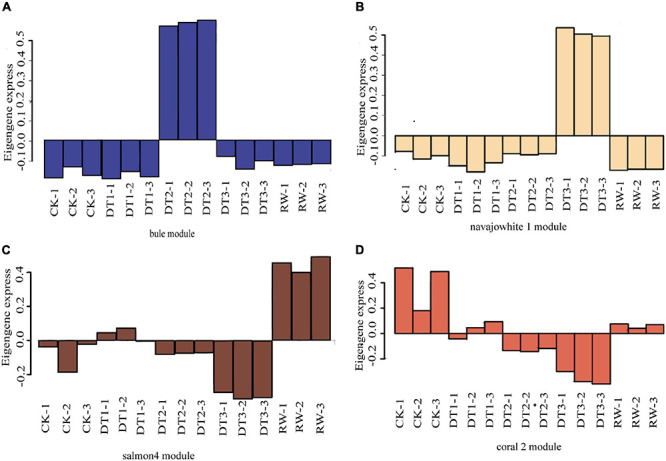
The expression pattern of the co-expressed genes in the representative module. **(A)** Blue module. **(B)** Navajowhite1 module. **(C)** Salmon4 module. **(D)** Coral2 module.

Gene co-expression network gene clustering and module cutting combined genes with similar expression patterns on the same branch. Each branch represented a co-expression module with different colors representing different modules. The unexpressed genes in more than half of the samples were filtered, the expression patterns of 30,012 genes obtained from transcriptome sequencing were performed by WGCNA. A total of 18 modules were identified according to the similarity of expression patterns (Figure 5A). Cluster analysis was performed to evaluate genes in the modules and were observed through heat maps (Figure 5B). Furthermore, to identify modules that were significantly associated with different drought stress, the module-trait correlation relationships were constructed (Figure 5C). There were four modules including blue, navajowhite1, salmon4, and coral2 were closely associated, with traits related to four treatments (0.99, 0.99, 0.87, and 0.76, respectively). The expression pattern of the eigengenes represents the gene expression profile of the entire module, and the expression patterns of the four selected modules were analyzed (Figure 6).

**FIGURE 7:**
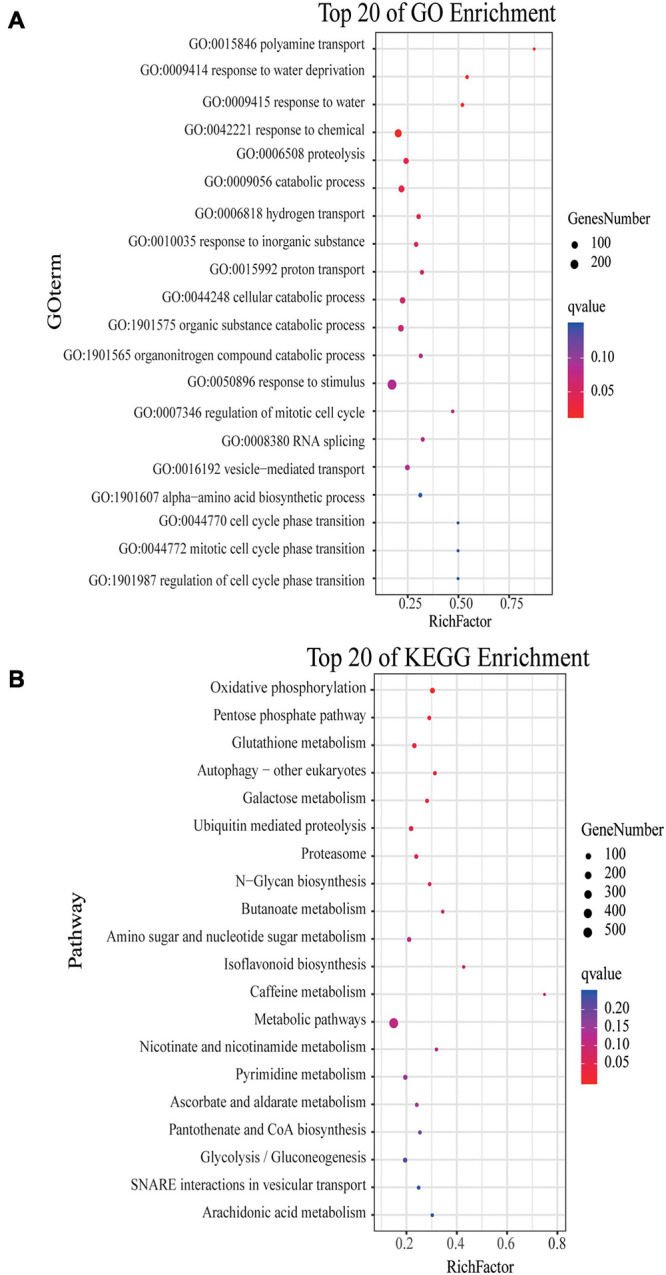
GO and KEGG analysis of DEGs in the navajowhite1 module. **(A)** The top 20 of GO enrichment. **(B)** The top 20 of KEGG enrichment. (The *q*-value ranges from 0 to 0.05. The closer the *q*-value is to 0, the more significant the enrichment is. Genes number is the number of genes enriched in pathways).

Four modules of blue, navajowhite1, salmon4, and coral2, were annotated through GO and KEGG analysis. The blue module of DT1 was most significant in GO analysis, which was mainly enriched in signal transduction and stress response (Supplementary Table 13). The results of KEGG analysis were similar, mainly focusing on signal transduction and synthesis of metabolites (Supplementary Table 13). The navajowhite1 module showed significant rich GO terms, indicating many genes were enriched in various stress responses. Those that were mainly enriched in ‘response to stimulus’ (GO:0050896), ‘response to chemical’ (GO:0042221), ‘response to water’ (GO:0009415), and ‘response to water deprivation’ (GO:0009414) were also significantly enriched (Figure 7A). KEGG enrichment analysis displayed the main enrichment in metabolic pathways (Figure 7B). In the salmon4 module, GO terms and KEGG pathways were enriched in metabolic progress, which indicates that the rose produces metabolites in the later drought stage to resist drought stress (Supplementary Tables 13, 14). However, the process of compound metabolisms was focused on the coral2 module (Supplementary Tables 13, 14). The GO terms about stress response are mainly concentrated in navajowhite1 and blue module. As a result, navajowhite1 and blue module genes may play a critical role in the process of rose drought stress and rewatering.

### Identification of Hub TFs and Network Construction

**FIGURE 8:**
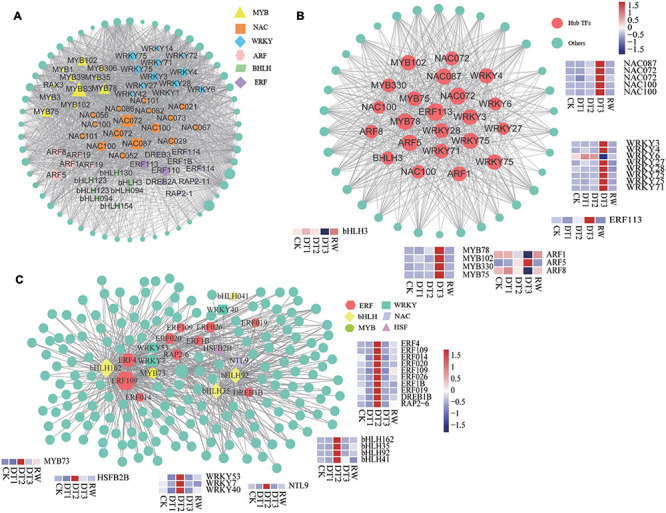
Identification and selection of vital transcription factors in navajowhite1 and blue module. **(A)** Network analysis of TFs in navajowhite1 module. **(B)** Network of the top 22 hub TFs and related genes in the navajowhite1 module. **(C)** Network analysis of hub TFs in the blue module.

Transcription factors are an essential class of regulatory proteins in biological processes. This study analyzed the TFs in each module. Although there was a difference in the distribution of TFs in different modules, it is mainly concentrated in *WRKY*, *NAC*, *MYB*, *ERF*, *bHLH*, *bZIP*, *C2H2*, and other transcription factor families. According to previous reports, these TFs were involved in the process of plant stress regulation (Erpen et al., 2018). TFs of navajowhite1 and the blue module were analyzed, the highly connected examples that were used as core genes were screened out. Many DEGs Identified in the navajowhite1 module were annotated as TFs, including several *WRKY*, *MYB*, *NAC*, *ERF*, *ARF*, and *bHLH* TFs (Figure 8A). Based on the hub gene correlation network and their high connectivity, 22 of these TFs were selected: two genes (RchiOBHm_Chr5g0034761 and RchiOBHm_Chr6g0308271) encoding *NAC072*, two genes (RchiOBHm_Chr2g0167461 and RchiOBHm_Chr7g0181811) encoding *NAC100*, *NAC087* (RchiOBHm_Chr3g0497471), *MYB33* RchiOBHm_Chr7g0228621), *MYB75* (RchiOBHm_Chr2g0116041), *MYB102* (RchiOBHm_Chr5 g0006031), *MYB78* (RchiOBHm_Chr4g0426181), *WRKY3* (RchiOBHm_Chr4g0439041), *WRKY4* (RchiOBHm_Chr2 g0166991), *WRKY6* (RchiOBHm_Chr5g0040801), *WRKY27* (RchiOBHm_Chr1g0378621), *WRKY28* (RchiOBHm_ Chr2g0151681), *WRKY71* (RchiOBHm_Chr5g0013131), two genes (RchiOBHm_Chr4g0429851 and RchiOBHm_ Chr2g0175911) encoding *WRKY75*, *ERF113* (RchiOBHm_Chr4g0428891), *ARF1* (RchiOBHm_Chr7g021 9771), *ARF5* (RchiOBHm_Chr6g0302551), *ARF8* (RchiOBHm_Chr3g0487771), and the *bHLH3* (RchiOBHm_Chr6g0288981) (Figure 8B). The experiment showed the expression of these genes was significantly upregulated at DT3 except for *WRKY6*, *bHLH3*, *ARF1*, and *ARF8* (Figure 8B). In the blue module, 20 TFs were identified as hub genes including *ERFs*, *bHLHs*, *WRKYs*, *MYBs*, and *NACs* (Figure 8C). *ERF109* and *bHLH162* have higher connectivity among them (Figure 8C). The expression of all 20 TFs increased in DT2 (Figure 8C).

### Validation of the DEGs by qRT-PCR

**FIGURE 9:**
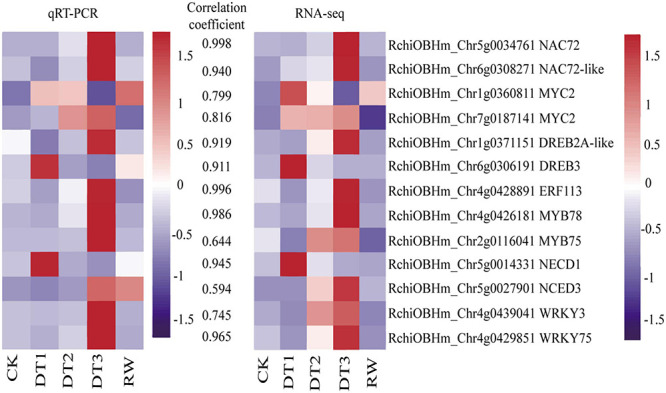
Heatmaps of the validation and correlation analysis of 13 selected DEGs. The values between the two heatmaps represent the correlation coefficients of qRT-PCR and RNA-seq values from each gene.

To verify the accuracy and reproducibility of the transcriptome analysis, 13 DEGs were selected to analyze the transcript abundance using qRT-PCR, including seven randomly selected transcription factors from the navajowhite1 module, four TFs, and two ABA synthesis-related DEGs. The results showed that the expression profiles detected by qRT-PCR were positively correlated with the RNA-Seq results (Figure 9). Therefore, the reliability of the RNA-seq data was confirmed by the consistency between the qRT-PCR results and RNA-seq analyses.

## Discussion

### TFs Involved in the Drought Stress Response

Weighted gene co-expression network analysis is an analysis method for analyzing the gene expression patterns of multiple samples. Genes with similar expression patterns can be clustered, moreover, the relationship between modules and specific traits can be analyzed (Zhang and Horvath, 2005). WGCNA analysis can present the interaction relationship between genes, and identify the hub genes at the center of the regulatory network. It also can use the module eigengenes values to perform correlation analysis with specific traits, more accurately (Silva et al., 2019). More and more studies use WGCNA to analyze key genes in plants under abiotic stress (Shao et al., 2021). In this study, through the analysis of WGCNA and transcription regulation modules, the highly connected TFs in modules were selected. Numerous TFs were obtained in the transcriptome sequencing in this study including the *NACs*, *WRKYs*, *MYBs*, *AP2/ERFs*, *bHLHs*, and so on. Many studies have shown that *NACs* play positive roles in the regulation of plant stress resistance. In particular, Arabidopsis (Tran et al., 2004), wheat (Mao et al., 2014; Zhang et al., 2015), and rice (Hong et al., 2016; Sung et al., 2018) have received the most research attention on *NACs*, which found that overexpression of *NACs* transcription factor family genes can significantly enhance the tolerance of transgenic plants to multiple stresses. In addition, rose (*Rosa hybrida*) *RhNAC3* can improve rose petals dehydration tolerance, and can also improve the drought tolerance of transgenic Arabidopsis (Jiang et al., 2014). In the study of rose (*Rosa chinensis* ‘Slater’s crimson China’) heat resistance, it was found that both *NAC27* and *NAC72* up-regulated (Li et al., 2019b). *NAC27* and *NAC72* were also involved in ABA signal transduction under heat stress of *Rhododendron hainanensis* (Zhao et al., 2018). Overexpression of *NAC72* in Arabidopsis thaliana can increase the drought resistance of plants, and also proved that it mediates the regulation of ABA-responsive genes (Li et al., 2016). Gene encoding *NAC072* with high connectivity in the navajowhite1 module was selected, indicating that it plays an important role in the regulation of rose drought stress. In agreement with previous studies, it can be concluded that *NAC072* may be involved in ABA signal transduction under drought stress in *R. chinensis*. Furthermore, the role of the core gene in the navajowhite1 module *NAC087* and *NAC100* in drought also requires further consideration.

The *WRKY* genes play pivotal roles in stress responses. Many researchers have demonstrated that *WRKYs* have crucial biological functions in the response of plants to different kinds of biotic and abiotic stresses (Jiang et al., 2017). Studies on Arabidopsis, rice, and soybean have shown the importance of the *WRKYs* in response to drought stress. For example, *AtWRKY57* (Jiang et al., 2012), *AtABO3* (Ren et al., 2010), *OsWRKY47* (Raineri et al., 2015), and *GmWRKY54* (Wei et al., 2019) can improve the drought tolerance of plants. Likewise, *VaWRKY14* responds to drought and cold stress, and hence the overexpression of *VaWRKY14* can enhance the drought tolerance of transgenic Arabidopsis (Zhang et al., 2018). However, some *WRKYs* can act as a negative regulator of abiotic stress in plants. For instance, *GhWRKY33* (Wang et al., 2019b) and *SlWRKY81* (Ahammed et al., 2020; Li et al., 2020) reduce the drought tolerance of transgenic plants. In the analysis, eight *WRKY* genes including *WRKY75*, *WRKY28*, and *WRKY27* were found to have high connectivity in the navajowhite1 module, indicating that they may be involved in drought response. Other research has found that *PagWRKY75* was down-regulated in the early stages of salt and osmotic stress, transgenic poplar lines overexpressing *PagWRKY75* were more sensitive to salt and osmotic (Zhao et al., 2019b). It can be assumed that *WRKY75* plays an essential regulatory role in abiotic stress, but the function of *WRKY75* in drought stress screened in this study requires further verification.

APETALA2/ethylene-responsive factor (*AP2/ERF*) TFs have been found to regulate plants and to enable them to be resistant to abiotic stress (Li et al., 2021b). In this study, AP2/ERF TFs were observed that may be involved in the regulation of rose drought stress. One gene encoding *ERF109* identified in a blue module appeared to play an important role in roses in resisting drought stress, the function of which requires further study. The study showed that *PtrERF109* is positively regulated with *Poncirus trifoliata* to resist the cold (Wang et al., 2019a). Moreover, *DREB2A*, *DREB3*, and *DREB1B* were found in this study. More and more studies have shown the function of *DREBs* in resisting abiotic stress in plants. Overexpression of *StDREB2* improved the drought resistance of cotton (El-Esawi and Alayafi, 2019). However, *RhDREB2B* is a negative regulator to resist abiotic stress (Li et al., 2021b).

The function of *bHLHs* in resisting abiotic stress in *Arabidopsis thaliana* has also been studied (Liu et al., 2014; Qiu et al., 2020). *bHLH162* and *bHLH35* have been identified in this study, providing new candidate genes for roses to resist drought stress. Meanwhile, *MYBs* including *MYBS3*, *MYB75*, and *MYB78* require further investigation. Particularly for rice, *OcMYBS3* is essential for resisting cold stress (Su et al., 2010).

### Phytohormone Signals Under Drought Stress

Phytohormone plays a key role in abiotic stress responses and coordinates various signal transduction pathways. In this study, the gene expression involved in several plant hormone-related signal transduction pathways changed significantly after drought treatment, indicating that plant hormones may play a crucial role in the response of the rose to drought stress. ABA acts as an endogenous messenger in a plant’s abiotic stress responses (Teng et al., 2014). Drought results in a substantial increase in plant ABA levels, accompanied by a major changes in gene expression and adaptive physiological responses. NCED is a key rate-limiting enzyme in the ABA synthesis pathway (Schwartz et al., 1997; Iuchi, 2002). Currently, some *NCED3* genes have been isolated and identified, corresponding gene function studies have also shown that *NCED3* genes play an important role in enhancing plant drought resistance (Li et al., 2019a). The study found that an *NCED3* gene was up-regulated by 2.70-fold at DT1. Interestingly, *NCED6* down-regulated by 9.40-fold in DT3. Lefebvre et al. (2006) state that *AtNCED6* is involved in ABA biosynthesis during seed development. The role of *NCED6* in drought stress still requires further study. Another ABA synthesis related to gene aldehyde oxidase (*AAO3*) and zeaxanthin cyclooxygenase (*ZEP*) were identified, both of which were significantly down-regulated in DT3. These results suggested that the synthesis of ABA may increase during the early drought stage and decrease during the later drought stage. Therefore, ABA-mediated signal transduction may be involved in the drought response of *R. chinensis*.

There is more and more evidence suggesting that the phytohormone ethylene is essential for regulating various developmental processes and stress responses of plants (Pei et al., 2017). Mcmichael et al. (1972) reported that the release of ethylene may increase under drought conditions. Studies on pineapples have shown that drought-stressed plants produce significantly less ethylene in leaf and stem tissues compared to control plants (Min and Bartholomew, 2005). These conflicting results may be due to the differences in distinct species. In general, the production of ethylene can be affected by changing *ACC* biosynthesis under drought stress (Larrainzar et al., 2014). In plants, ACC synthase (*ACS*) and ACC oxidase (*ACO*) are the two most essential enzymes in the ethylene biosynthetic pathway (Kende, 1989). In the study, the expression of three *ACS* genes was suppressed under drought stress, the expression of one *ACS* gene up-regulated, while the expression of two *ACO* genes was also up-regulated. It is not yet possible to speculate whether the ethylene content has increased or decreased under drought conditions. Ethylene response factors (*ERFs*) are downstream components of the ethylene signaling pathway (Guo and Ecker, 2004). Experiments showed that *ERFs* play important roles in plant abiotic stress response (Pan et al., 2012; Yu et al., 2017). A large number of *ERFs* exhibit different expressions under different drought levels. Their role under drought stress needs further study.

Jasmonates (JAs), a class of oxygenated lipid derivatives, are phytohormones necessary for plant growth and environmental adaptation (Wasternack and Hause, 2013). *MYCs* proteins are one of the JASMONATE-ZIM (JAZ) target proteins and a key transcription factor regulating the corresponding genes downstream of JA. According to the reports, *MYC2*, *MYC3*, and *MYC4* are the central nodes of the JA signal regulation (Chico et al., 2020). Lipoxygenase (*LOX*), allene oxide synthase (*AOS*), allene oxide cyclase (*AOC*), and oxophytodienoate reductase (*OPR*) are important enzyme genes in the JA synthesis pathway (Devoto and Turner, 2005). The study has observed that *RhHB1* binds to the *RhLOX4* promoter to suppress its expression in the cut rose (*Rosa hybrida*). As a result, dehydration tolerance decreases (Fan et al., 2020). These genes were identified, while most genes were up-regulated under drought stress. Other studies have shown that JA was observed to increase in *Pinus pinaster* (Pedranzani et al., 2007) plants and *Oryza sativa* (rice) (Du et al., 2013) leaves exposed to drought conditions. Based on these results, it can be conjectured that the JA pathway plays a role in the response to drought stress in *R. chinensis*.

Moreover, recent studies have indicated that the role of brassinosteroid (BR) in plant response to drought stress (Nolan et al., 2017a, b, 2020). In Fabregas et al.’s (2018) study, the overexpression of *BRL3*, a vascular-rich member of the BR family, can confer Arabidopsis drought resistance without compromising normal plant growth. In this study, the expression of two *BRL* genes increased under drought stress, and hence BRLs may play positive parts in the response to drought. In addition, BR and ABA regulated the drought resistance of plants. Indeed, the molecular basis of the antagonistic effect of the BR-ABA pathway has been defined (Wang et al., 2018; Nolan et al., 2020). The role of ABA in promoting the response to drought stress suggests that BRs will inhibit drought stress responses. Yet, only the role of hormone series resistance has been explored in model plants. This study also laid the foundation for the interaction of hormones in the drought resistance of rose.

### Signaling Pathway Mediates Drought-Stress Responses

When plants are subjected to adversity stress due to changes in the external environment, they sense adversity stimuli from outside, recognize and transduce between cells and cells, and then transmit them to downstream genes, thereby causing downstream affect gene expression to resist adversity. Calcium ions are the second messengers of the cell, which mediates the calcium signal plays an essential role in the plant response to external stimuli (Kudla et al., 2010; Reddy et al., 2011). As a second messenger, calcium ion can not only maintain the stability of cell structure, but also play a vital role in signal transmission and participate in plant stress response (Xiong et al., 2002; Shinozaki et al., 2003). The three main families of calcium sensors in Ca^2+^ signaling in plants are calmodulin (*CaM*), calcineurin B-like (*CBL*), and calcium-dependent protein kinases (*CDPKs*) (Ranty et al., 2006; Boudsocq and Sheen, 2013). Analysis of the *CaM/CMLs* identified in this study revealed that five genes up-regulated, two up-regulated and then down-regulated, and fifteen down-regulated under drought stress. *AtCML8*, *AtCML13*, *AtCML18* and *AtCML25* down-regulated by salt damage and drought (McCormack et al., 2005). *ShCML44* up-regulated under cold, drought, and salt damage (Munir et al., 2016). In contrast, the gene encoding *CML44* down-regulated and the gene encoding *CML8* up-regulated under drought induction in this study. *CDPKs/CPKs* are involved in multiple stress signaling pathways. In Arabidopsis, Overexpressed *AtCPK6* confers drought tolerance (Xu et al., 2010), while *AtCPK21* is a negative regulator of osmotic response (Franz et al., 2011). In this study, six genes encoding putative Ca^2+^-dependent protein kinases up-regulated during drought stress, and four of them showed high expression levels during the DT2 period. These results indicate that the CDPK pathway was significantly activated in DT2. CBLs communicate with downstream CIPKs for signal transmission. The CBL-CIPK pathways also participate in plant abiotic stress response (Xiang et al., 2007). In the current research, three genes encoding *CBLs-CIPKs* significantly down-regulated in DT3, which indicates that the CBL-CIPK pathway may contribute to regulating a later drought response in *R. chinensis*. In summary, the above results suggest that Ca^2+^ mediated signaling pathway has key functions in the response of *R. chinensis* to drought stress.

Plant’s sense and mediate various intracellular and extracellular signals through cell surface receptor kinases after stimulating by the external environment. Studies have shown that receptor-like protein kinases (RLKs) play a vital role in plant growth and stress adaptation (Wang et al., 2007). Leucine-rich repeat receptor protein kinases (LRR-RLKs), a class of single transmembrane proteins, are the largest family of plant receptor protein kinases (Gou et al., 2010). The *LRR-RLKs* genes identified by the study were 1 up-regulated and 10 down-regulated under drought stress, which was similar to the expression of *LRR-RLKs* genes (3 up-regulated, 38 down-regulated) in chrysanthemum under dehydration stress (Xu et al., 2013). Additionally, multiple *MAPKs* were observed during the plant response to abiotic stresses such as salt, drought, cold, and heat, mainly rapid activation of *MPK3*, *MPK4*, and *MPK6* (Zhu, 2016). The results in this study are similar, as gene encoding *MPK3* significantly down-regulated on DT1, indicating that *MPK3* responded quickly when drought stress occurred.

MAPK cascade signals can also transmit reactive oxygen species (ROS) signals to downstream targets. When plants were subjected to environmental stress, ROS generated signals in the plant, triggering a series of changes. Plants also have an antioxidant system protecting against poisonous oxygen (Jaspers and KangasjcTrvi, 2010). Abiotic stress can cause plants to produce excessive ROS which can cause damage to plants. Plants have efficient enzymes including SOD, POD, APX, GPX, and so on. The antioxidant defense mechanisms can protect plants from oxidative stress (Gill and Tuteja, 2010). In this study, two genes encoding *APX* were up-regulated and four were down-regulated. Similarly, in the study of *Citrullus lanatus*, the changes of *APX* genes are different under drought stress (Goitseone et al., 2018). The post-harvest petals of cut rose (Rosa *hybrida* cv. Samantha) under water deficit stress reported that the Transcription level of *RhAPX* may be involved in the response of water deficit stress (Jin et al., 2006). Interestingly, three genes encoding *SOD* were identified, all of which were reduced under drought stress. When SOD activity was measured, the level of SOD activity increased as the degree of drought stress increased. The expression pattern of SOD genes in this study is similar to the expression pattern of the *SmFSD2* in drought. The *SmFSD2* did not change significantly over 3 days, but decreased to the lowest value at 9 days (Shafi et al., 2019). These results indicate that the ROS signaling pathway may be involved in the regulation of *R. chinensis* response to drought stress.

### Metabolism in Response to Drought Stress in *R. chinensis*

Primary and secondary metabolites act as signaling molecules or protective agents when plants respond to adversity stress. The primary metabolic processes were involved in the response to the drought stress of *R. chinensis*, producing a series of osmotic protective agents. Such as sugars, starches, amino acids, and lipids. Accumulation of sugars in various plants is related to high tolerance to drought stress (Ali et al., 2019), which is consistent with the observation of the experiment. Most genes related to carbohydrate synthesis including fructose, glucose, mannose, sucrose, and trehalose were induced and significantly up-regulated under drought stress. The gene encoding trehalose phosphate synthase (*TPP1*) down-regulated under drought stress. Among the 11 *OsTPS* genes in the rice (*Oryza sativa*) genome, only *OsTPS1* has TPS activity (Zang et al., 2011). Overexpression of *OsTPS1* can improve the tolerance of rice seedlings to drought stress. At the same time, it also causes the expression of some stress-related genes to be up-regulated and the phenotype has no obvious change (Li et al., 2011). Trehalose may involve in the process of rose’s resistance to drought. The expression levels of the four genes encoding starch synthase significantly reduced at DT3, with no significant change in expression levels in the remaining periods compared with the control. It is speculated that the starch content may decrease in a later drought.

Lipids are essential components of cells and organelles. A series of genes that synthesize lipid biosynthesis have been identified in drought treated *R. chinensis* plants. Long-chain acyl-CoA synthetases (LACS) are indispensable in the pathway of lipids synthesis and degradation of higher plants. There are nine genes in the *LACSs* family in Arabidopsis, among which *LACS1* and *LACS2* are involved in the synthesis of wax and cutin together, with an overlap in function (Lü et al., 2009; Weng et al., 2010). Drought stress can cause rapid accumulation of wax on plant surface (Kosma et al., 2009). In this study, six genes encoding *LACS2* were induced under drought, while five of them significantly down-regulated at the DT3. Studies on bananas, *Agave sisalana* (Sarwar et al., 2019), and *Poa pratensis* (Ni et al., 2016) have reported that waxy synthetic transcripts significantly down-regulated under drought stress. These results confirm that wax may play key roles in regulating the drought resistance of *R. chinensis*. In brief, it can be speculated that the wax content may be reduced in the later period of drought stress. In addition, the gene encoding GDSL esterase/lipase was significantly induced at the beginning of drought stress. Studies on the GDSL esterase/lipase gene of *Arabidopsis thaliana* revealed that this gene helps plants resist abiotic stress (Lai et al., 2017), and is induced in *M. wufengensis* by cold stress (Deng et al., 2019). In summary, lipid metabolism is crucial for *R. chinensis* adaptation to drought stress.

Furthermore, as part of the adaptation mechanism to the environment of plants, secondary metabolism is sensitive to both biological and abiotic stresses (Hartmann, 2007). It is known that the phenylpropane pathway has high responsiveness to different abiotic stresses such as injury, drought, and low or high temperature. White grapes (*Vitis vinifera* L.) cope with drought by stimulating the phenylpropane pathway, which reflects the result of this study. The experiment showed that most of the secondary metabolites are derived from phenylpropane biosynthesis and flavonoid biosynthesis pathways. The genes that encode phenylalanine ammonia lyase (*PAL*) and coumaric acid-CoA ligase (*4CL*) were identified. They are all related to enzyme genes in the phenylpropane metabolism pathway. They significantly up-regulated at DT1, indicating that the phenylpropane metabolism pathway was induced under drought stress. The transcription of *PAL* of *Caragana korshinskii* increased under field and laboratory drought conditions (Liu et al., 2019). These results also confirm the results of the present report. Another major derivative of the phenylpropane pathway is flavonoids, which play a key role in adversity stress. Flavonoids with free radical scavenging activity alleviate oxidative and drought stress in *Arabidopsis thaliana* (Nakabayashi et al., 2014). This study reported that genes encoding flavonoid synthase (*FLS*), chalcone isomerase (*CHS*), and anthocyanin synthase (*ANS*) were induced under drought. Flavonoid products may affect the regulation of *R. chinensis* by drought stress.

### Model Construction of Rose Drought Response

**FIGURE 10:**
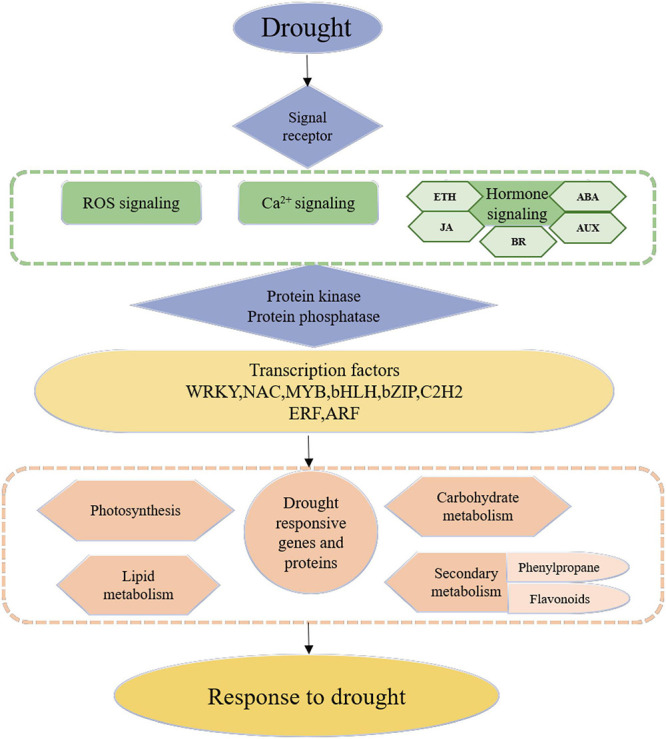
The hypothetical model of *Rosa chinensis* ‘Old Blush’ in response to drought stress.

This study aimed to enhance understanding of the mechanisms through which *R. chinensis* responds to drought and identify the TFs related to drought tolerance. Figure 10 summarizes the model of *R. chinensis* drought response. According to the transcriptome data, signal receptors, such as ion channel proteins sense external drought stimuli and transmit through Ca^2+^, ROS, and phytohormone signaling transduction. The signal transmits protein kinases and protein phosphorylases, which can activate the transcription factors. The activation of TFs triggers downstream drought-responsive gene transcription such as lipid metabolism, carbohydrate metabolism, and secondary metabolism to regulate cell homeostasis. The hub TFs involved in roses’ drought response and rewatering were identified through WGCNA, providing resources for further research on the drought control network of roses. The new findings of this study, on the relationship between unreported TFs and drought resistance regulation, still require further experimental explore.

## Data Availability Statement

The datasets presented in this study can be found in the NCBI repositories (BioProject ID: PRJNA722055).

## Author Contributions

YL, SZ, XJ, and HF conceived and designed the research. XJ performed the experiments, analyzed the data, and wrote the manuscript. YB and NJ cultivated and provided the rose plants. All authors have read and agreed to the published version of the manuscript.

## Conflict of Interest

The authors declare that the research was conducted in the absence of any commercial or financial relationships that could be construed as a potential conflict of interest.

## Supplementary Material

Supplementary Figure 1 The distribution of cleaned RNA-Seq reads mapped to the rose reference genome. **(A)** Percent of mapped to genome regions of CK groups. **(B)** Percent of mapped to genome regions of DT1 groups. **(C)** Percent of mapped to genome regions of DT2 groups. **(D)** Percent of mapped to genome regions of DT3 groups. **(E)** Percent of mapped to genome regions of RW groups.

Supplementary Table 1 Top 20 enriched GO terms of up-regulated DEGs.

Supplementary Table 2 Top 20 enriched GO terms of down-regulated DEGs.

Supplementary Table 3 Significantly enriched gene pathways involving DEGs under the drought stress treatment.

Supplementary Table 4 Protein kinases-related genes that were differentially expressed in response to drought treatment.

Supplementary Table 5 Calcium-related genes that were differentially expressed in response to drought treatment.

Supplementary Table 6 ROS producing and scavenging system-related genes that were differentially expressed in response to drought treatment.

Supplementary Table 7 Differently expressed related to hormone metabolism and signaling in response to drought treatment.

Supplementary Table 8 Starch and sucrose metabolism-related genes that were differentially expressed in response to drought treatment.

Supplementary Table 9 Lipid metabolism process-related genes that were differentially expressed in response to drought treatment.

Supplementary Table 10 Photosynthesis-related genes that were differentially expressed in response to drought treatment.

Supplementary Table 11 Transcription factor families.

Supplementary Table 12 The primers sequences of selected genes used in qRT-PCR.

Supplementary Table 13 Top 20 enriched GO terms of modules.

Supplementary Table 14 Top 20 enriched KEGG pathway of modules.
