# Supplementary figures and images for: Comparative Transcriptome and Weighted Gene Co-expression Network Analysis Identify Key Transcription Factors of Rosa chinensis ‘Old Blush’ After Exposure to a Gradual Drought Stress Followed by Recovery
Source: Front Genet. 2021 Jul 15;12:690264. doi: 10.3389/fgene.2021.690264 (PMC8320538; doi:10.3389/fgene.2021.690264)

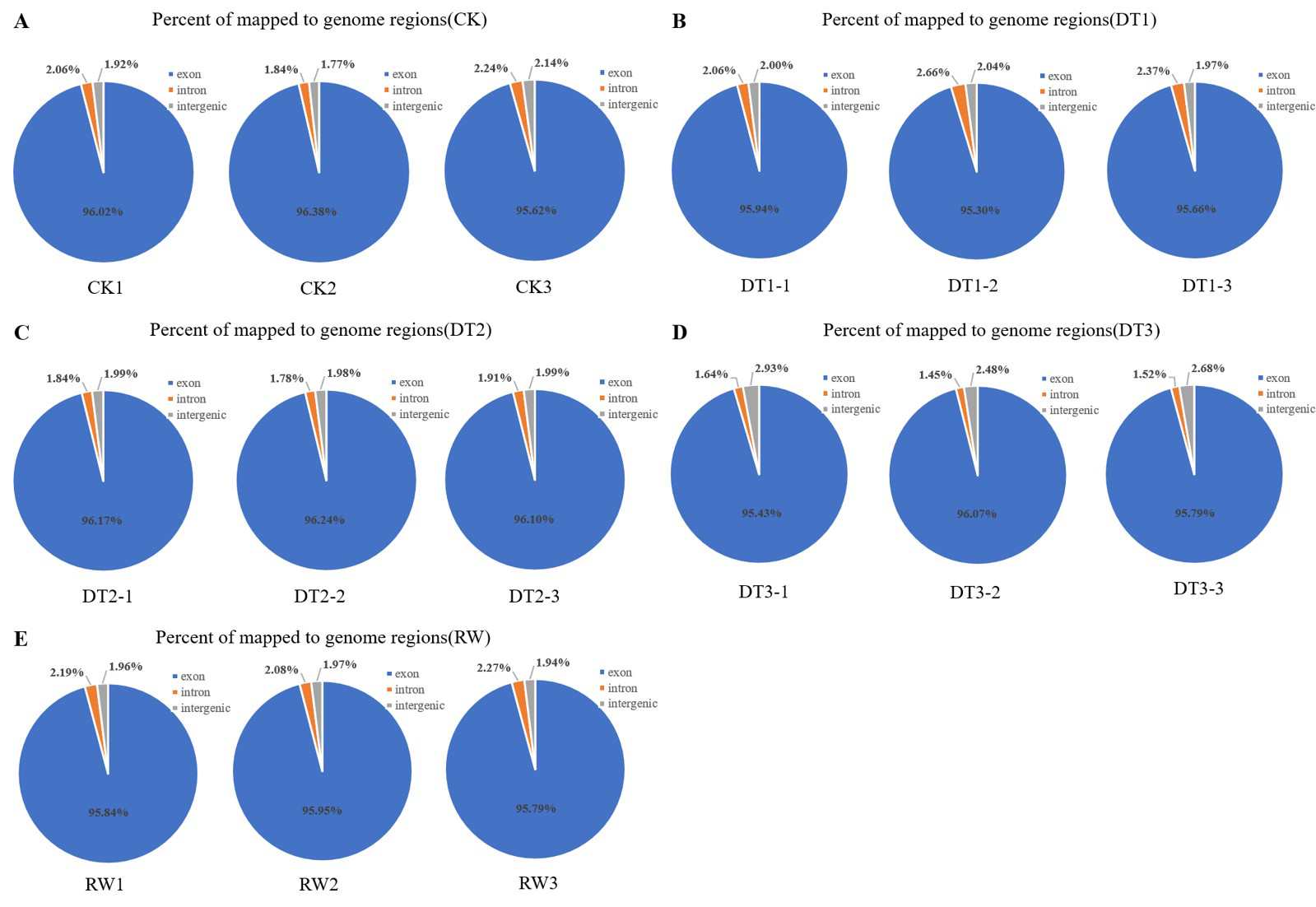

Supplement: Supplementary Figure 1 — The distribution of cleaned RNA-Seq reads mapped to the rose reference genome. (A) Percent of mapped to genome regions of CK groups. (B) Percent of mapped to genome regions of DT1 groups. (C) Percent of mapped to genome regions of DT2 groups. (D) Percent of mapped to genome regions of DT3 groups. (E) Percent of mapped to genome regions of RW groups. [file Image_1.TIF]
